# Evaluation of Family Physicians’ Diagnostic and Therapeutic Approach to Different Dermatological Diseases

**DOI:** 10.5152/eurasianjmed.2025.25778

**Published:** 2025-06-13

**Authors:** Ecem Bostan, Mahmut Talha Uçar

**Affiliations:** 1 Department of Dermatology and Venereology, Ankara Medipol University Faculty of Medicine, Ankara, Türkiye; 2 Dermatology and Venereology Clinic, Özel Bilgi Hastanesi, Ankara, Türkiye; 3 Department of Public Health, Health Sciences University Hamidiye Faculty of Medicine, İstanbul, Türkiye

**Keywords:** Dermatology, physicians, family, primary health care

## Abstract

**Background:**

Skin-related health problems constitute a considerable portion of the reasons for consulting a family physician. Therefore, family physicians play a key role as gatekeepers in evaluating the signs and symptoms of various skin diseases, triaging the patients, and deciding upon the necessity for referral. The aim was to investigate the most common dermatological diseases encountered by family physicians in the outpatient clinics and determine the diagnostic and remedial approach of family physicians to these skin diseases in Türkiye.

**Methods:**

An online questionnaire composed of 48 questions related to the demographic and educational information of the participants, the most frequently seen dermatoses in outpatient settings, and the management skills of the participants for various skin disorders, was created using Google Forms. Via instant messaging and e-mail, the survey was distributed among practitioner family physicians, family physician residents, and family medicine specialists who were actively employed in different healthcare facilities in Türkiye. The snowball sampling method was used to convey the survey.

**Results:**

The present questionnaire-based study was conducted between October 2024 and January 2025. A total number of 176 participants who were actively working in different healthcare facilities in Türkiye were included in the study. The median number of patients examined in a month was 1025 (range: 90-4000). The most commonly encountered cutaneous diseases were fungal infections of the hair, nail, skin, and mucous membranes (91.5%) followed by scabies (80.1%), acne vulgaris (72.2%), and herpes simplex infection (65.9%). Herpes simplex infections, fungal infections of the skin, hair, nails, mucosa, and scabies were the 3 leading skin diseases at which family physicians felt competent while administering treatment.

**Conclusion:**

The results of the current study point out that since family physicians deal with a relatively high rate of skin disorders in their daily practice, it is quite essential to reinforce their diagnostic and therapeutic proficiencies through intensified dermatology courses and rotations.

**Main Points**

- In Türkiye, family physicians encounter a substantial number of patients with common skin diseases.
- The majority of the family physicians reported having difficulty in the management of malignant skin neoplasms, hidradenitis suppurativa, and psoriasis.
- Dermatologic education should be consolidated through undergraduate and postgraduate training interventions.

## Introduction

Primary healthcare plays a key role in the triage, management, and referral of patients who present with various medical conditions. Patients presenting for a previously undiagnosed health problem, as well as maintenance care and treatment of pre-existing medical conditions, first contact a primary care physician. Therefore, family medicine, being a very crucial medical specialty within the primary healthcare service in Türkiye, functions as a bridge between primary and specialist care. It is evident that family physicians serve as the gatekeepers of the health system, triaging patients and referring them to the relevant specialist physicians if necessary.

Skin problems compose a major percentage of the medical conditions for consulting to a family physician. A registry-based epidemiologic study conducted in the Netherlands showed that skin problems accounted for 12.4% of all diseases seen by family physicians.^1^ It was found that 65.1% of the patients who participate into the study, contacted only their family doctor for their skin-related problems whereas 1.6% chose to visit only a specialist within a specified time period.^1^ Another survey-based study from Denizli (a province of Türkiye) pointed out that out of all patients who contacted with primary care physicians; 19.5% and 11.5% presented with dermatologic problems during summer and winter periods, respectively.^2^ Furthermore, the majority of the general practitioners who participated into the study, stated that they have been experiencing difficulty while diagnosing skin disorders.^2^ Diagnostic uncertainty may lead to misdiagnosis, improper treatment and management of that particular dermatological problem. These findings once again underlie the fact that since primary care physicians encounter a wide range of dermatological disorders in the outpatient clinic, ongoing medical education related common skin diseases should be fostered and offered to them.

The present study aims to investigate the most common dermatologic problems dealt with by the family physicians, as well as their diagnostic and therapeutic approaches to these diseases in Türkiye. In addition, by inquiring about their status as a family doctor (practitioner, research assistant, or specialist) and the efficiency of the dermatology training they have undergone during their education period, the role of dermatology knowledge in clinical practice was intended to be explored.

## Materials and Methods

KTO Karatay University Faculty of MedicineThe present descriptive, questionnaire-based study included practitioners, residents, and family medicine specialists who have been actively working in various healthcare facilities in different provinces of Türkiye. An online survey consisting of 48 questions was generated using Google Forms (Google LLC, USA). The survey was distributed among the family doctors via the snowball sampling technique, between October 2024 and January 2025. The present study was approved by KTO Karatay University Faculty of Medicine ethical committee (Decision number: 2024/037, Date: September 26, 2024). Written informed consent was obtained from all individuals who agreed to complete the survey.

The survey consisted of 3 parts: (I) sociodemographical and educational information of the family physicians, (II) diagnostic modalities, (III) therapeutic modalities of the participants for common skin diseases (acne, allergic-irritant contact dermatitis, alopecia areata, atopic dermatitis, basal cell carcinoma, fungal infections of skin, hair, nail and mucous membranes, herpes simplex, hidradenitis suppurativa, drug eruption, malignant melanoma, seborrheic dermatitis, psoriasis, cellulitis/erysipelas, scabies, squamous cell carcinoma, viral wart, zona zoster). For parts II and III, participants’ responses were categorized using a Likert scale as follows: “1 = I have no knowledge about the disease,” “2 = I know the clinical features of the disease, but I can’t make the diagnosis,” “3 = I make the diagnosis with difficulty, but with no full confidence,” “4 = I make the diagnosis with some difficulty, but with confidence,” “5 = I can make the diagnosis confidently.” While analyzing the data, these answers were collected into 2 main categories in order to evaluate the participants’ competency in diagnosis more efficiently. In this context, answers 1, 2, and 3 were combined under “Category 1: Difficulty or incompetence,” while answers 4 and 5 were combined under “Category 2: Success or competence.”

The participants’ approaches to the treatment of cutaneous diseases were also categorized using a Likert scale as follows: “1 = I do not start any treatment, I refer the patient to the relevant specialist,” “2 = I administer the treatment with no confidence,” “3 = I administer the treatment with little confidence,” “4 = I administer the treatment with full confidence.” When analyzing the data, these 4 answers were collected into 2 main categories in order to more clearly evaluate the participants’ responses. In this context, answers 1 and 2 were combined under “Category 1: Incompetence or uncertainty,” while answers 3 and 4 were combined under “Category 2: Success or sufficiency.”

### Statistical Analysis

SPSS 23.0 (IBM SPSS Corp., Armonk, NY, USA) statistical program was used for data analysis. Descriptive statistics are given as number of units (n), percentage (%), mean, and median. The relationship between categorical variables was evaluated with the chi-square test and Fisher’s exact test. A value of *P* < .05 was considered statistically significant.

Since the study was conducted using a convenient sampling method, no prior sample size calculation was performed. However, a post hoc power analysis was conducted to evaluate whether the sample size was sufficient to detect meaningful differences.

For the chi-square test, the post hoc power analysis indicated a power of 97.8%, confirming that the sample size was adequate for detecting a medium effect size (Cohen’s w = 0.3) with an alpha level of 0.05. Additionally, the post hoc power analysis for Fisher’s Exact Test yielded a power of 76.2%, suggesting near-sufficient statistical power for detecting group differences. These results indicate that the study had sufficient power for categorical comparisons.

## Results

A total number of 176 family physicians were included in the present study. The median age of the family physicians who participated in the study was 35 years (minimum 26, maximum 67, mean: 38.7 ± 9.3), and 55.7% (n = 98) of the participants were women. The median number of years working as a family physician was determined to be 7 years (minimum 0.25 years, maximum 29 years, mean: 8.2 ± 5.8). The median number of patients examined in a month was 1025 (minimum 90, maximum 4000, average: 1115 ± 555). It was shown that 13% (median) of the patients examined by the family physicians within a month were admitted due to skin problems, and 75% (median) of the patients presenting with skin conditions were diagnosed and treated by the family physicians themselves.

The 3 most common healthcare institutions at which the participants were employed were family health centers (68.2%; n = 120), followed by research and training hospitals (11.4%; n = 20) and state hospitals (10.2%; n = 18). Sixty-seven (38.1%) participants were family physician residents registered in the “Contractual Family Medicine Specialization Training Program” (CFMSTP), 23.9% (n = 42) were general practitioners, 22.2% (n = 39) were family medicine specialists, whereas 15.9% (n = 28) were family medicine residents affiliated with the ‘Medical Specialization Program’ (MSP). Eighty-three (47.2%) reported completing a clinical dermatology rotation, while 51 (29%) did not complete any dermatology rotation during their medical speciality training whereas 42 (23.8%) did not go through any specialty training. Fifty-two (29.5%) family physicians found the dermatology rotation in their training program beneficial, 19 (10.8%) were undecided, whereas 12 (6.8%) thought that the dermatology rotation was inefficient. The sociodemographic, occupational, and educational characteristics of the participants were collectively summarized in Table 1.

**Figure 1.**
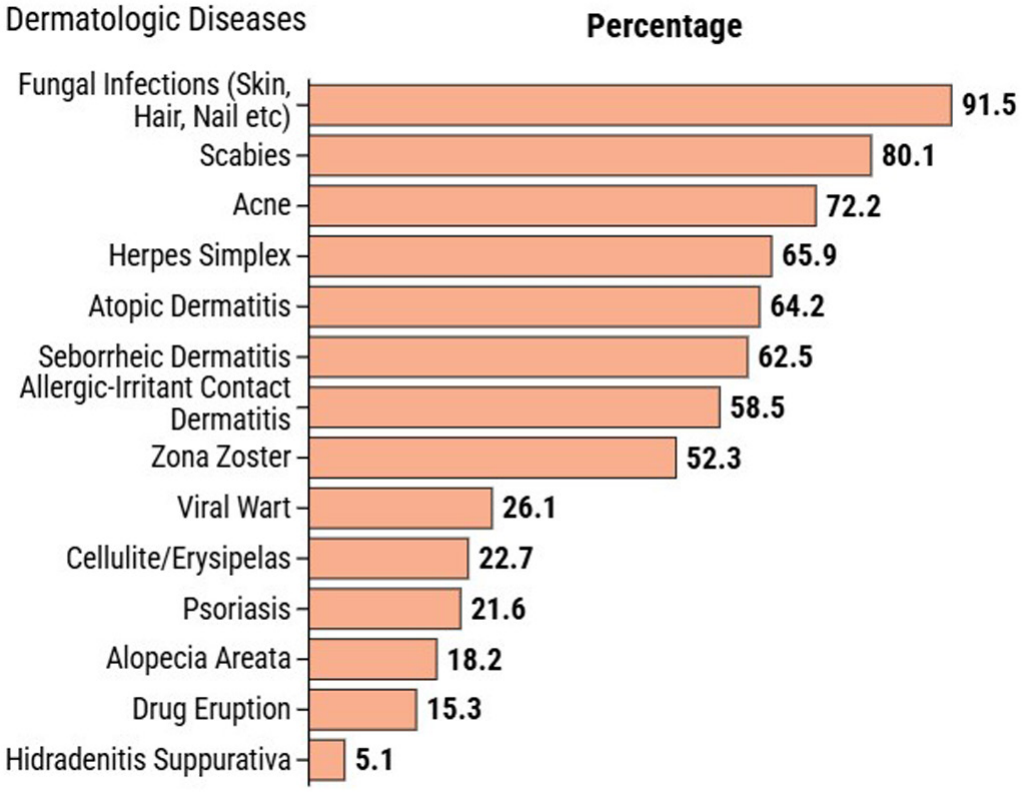
Percentage distribution of the most common skin diseases encountered by family physicians (multiple selections allowed).

**Figure 2.**
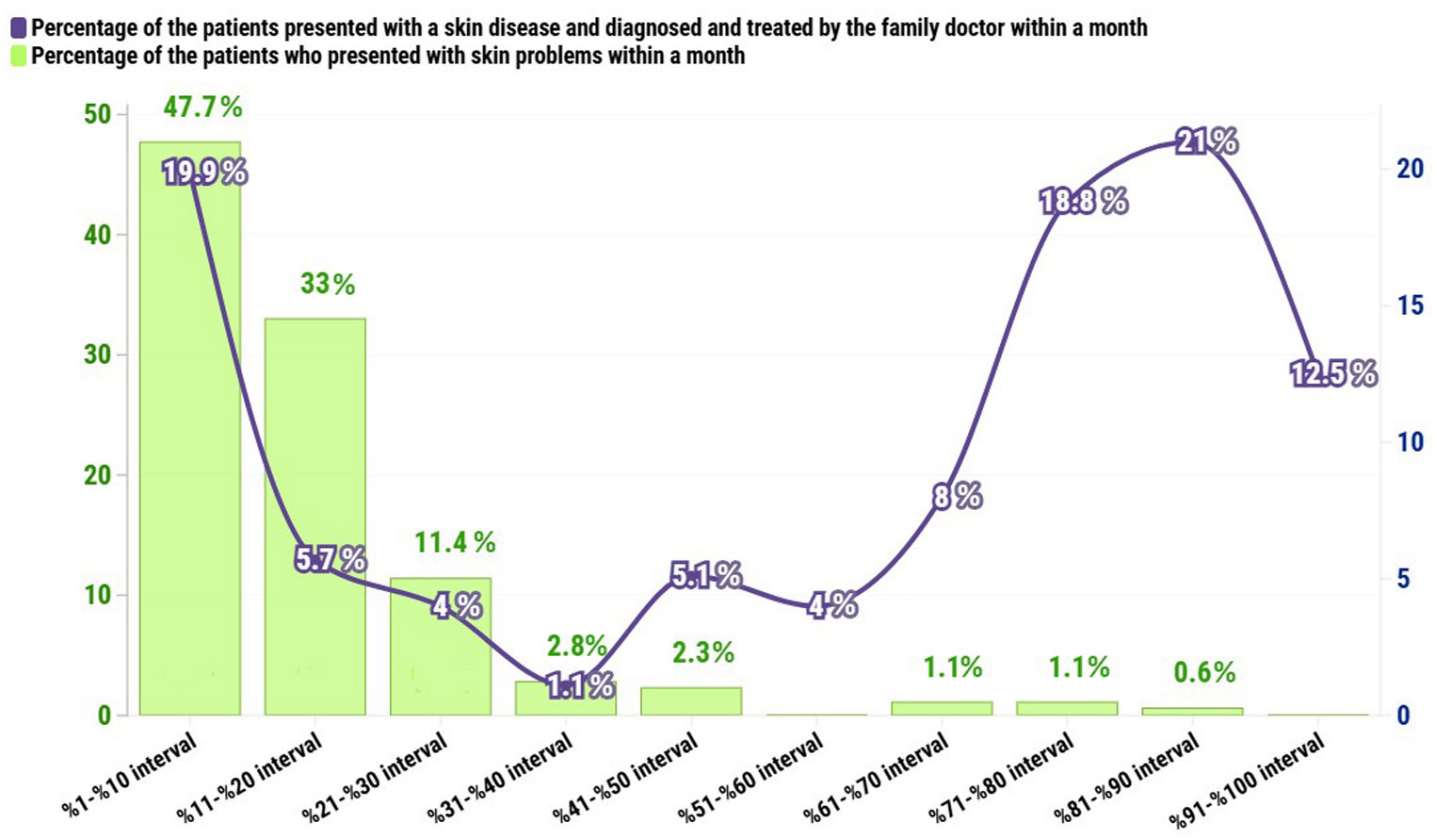
Percentage of patients who presented with skin problems within a month and those diagnosed and treated for a skin disease by family physicians within the same period.

**Figure 3.**
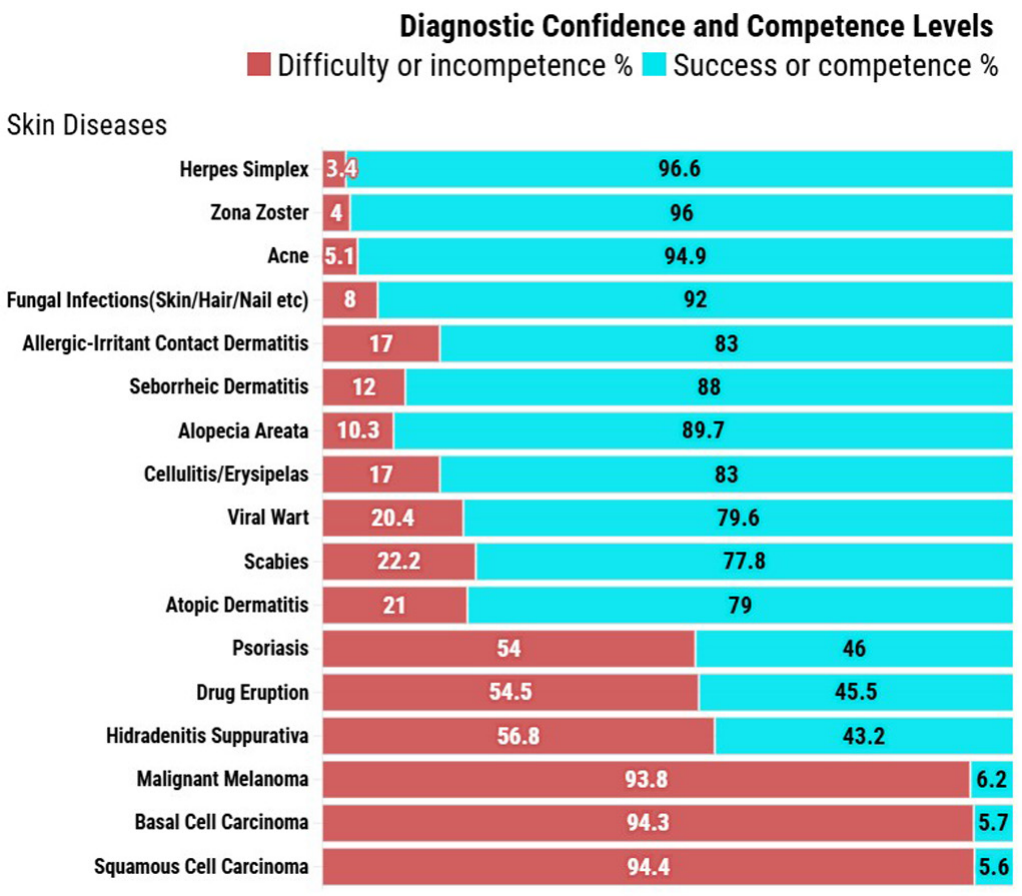
Diagnostic confidence and competence levels of the family physicians.

**Figure 4.**
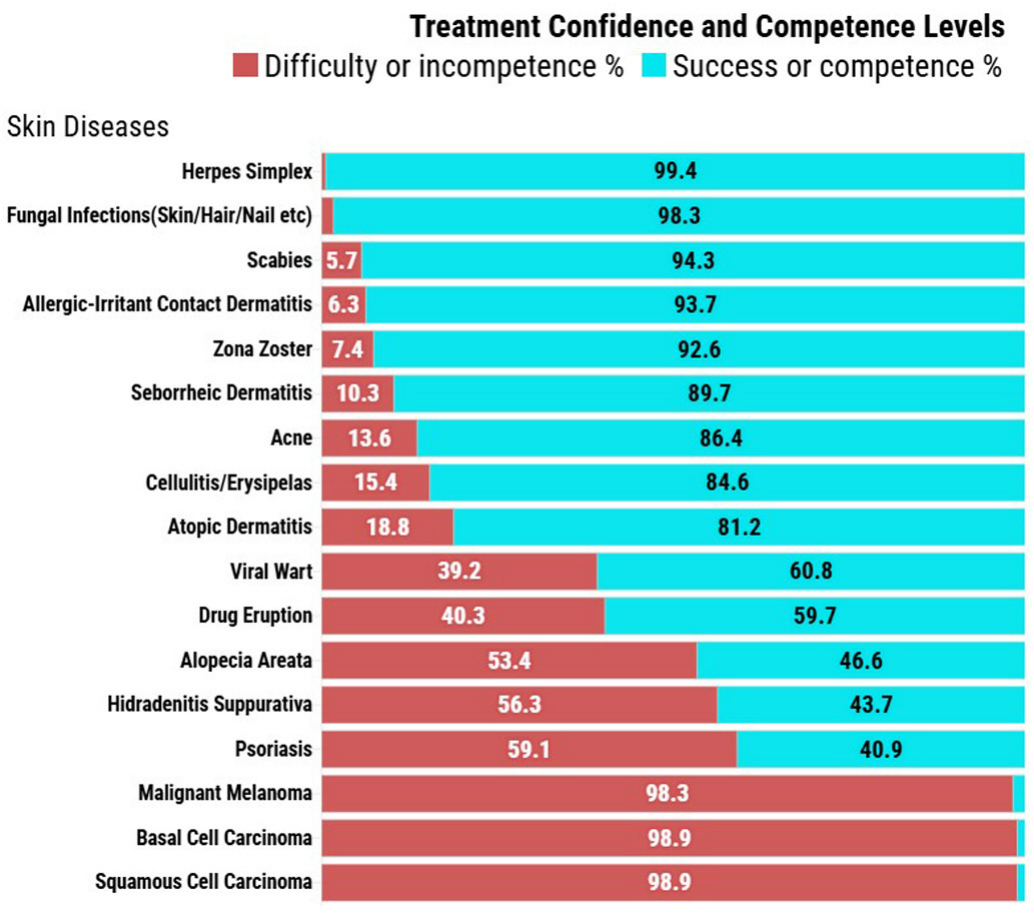
Therapeutic confidence and competence levels of the family physicians.

The distribution rates of cutaneous diseases seen by family physicians were based on the self-reports of the physicians. The most commonly encountered cutaneous diseases by family physicians in the outpatient clinics were fungal infections of the hair, nail, skin and mucous membranes (91.5%) followed by scabies (80.1%), acne vulgaris (72.2%) and herpes simplex infection (65.9%). Most of the family physicians stated that they did not have the opportunity to use potassium hydroxide for native preparation examination. The diagnosis was mainly based on clinical findings. The percentage distributions of the skin diseases encountered by the participants were shown in , Figure 1 whereas the percentage of patients who presented with skin problems within a month and those who were diagnosed and treated by the family physicians themselves within the same period were depicted in Figure 2. The diagnostic approaches of the family physicians to common skin disorders were summarized in Table 2 and given as a graph in Figure 3; whereas the management approaches of the family physicians to common cutaneous diseases were shown in Table 3 and depicted as a graph in Figure 4.

The participants’ competence in the diagnosis and treatment of various dermatological diseases was analyzed with respect to their dermatology rotation status. While 52.7% (n = 49) of the participants who did not undergo any dermatology rotation experienced difficulty or inadequacy in making the diagnosis of a drug eruption, this rate was determined to be 37.3% (n = 31) for those who had a clinical dermatology rotation. This difference was found to be statistically significant (Pearson chi-square = 4.162, *P* = .041). When evaluated in terms of treatment, 72% (n = 67) of the participants who did not have a dermatology rotation during their training found themselves adequate or successful when treating scabies, while this rate was found to be 86.7% (n = 72) for those who had a dermatology rotation. This difference was also found to be statistically significant (Fisher Exact Test, *P* = .020). Additionally, 76.3% (n = 71) of the participants who did not undergo a dermatology rotation, found themselves adequate or successful in treating cellulitis/erysipelas, while this rate was determined to be 94.0% (n = 78) for those who had a rotation. The difference between the 2 groups was found to be statistically significant (Pearson chi-square = 10.498, *P* = .001). In the treatment of atopic eczema, 75.3% (n = 70) of the participants who did not have a dermatology rotation, found themselves sufficient or successful, while this rate was found to be 88.0% (n = 73) for those who had a rotation. This difference was also found to be statistically significant (Pearson chi-square = 4.631, *P* = .031).

The diagnostic proficiency of the participants in dermatological diseases was also investigated according to their medical specialization status. No statistically significant difference was found between medical specialization status in terms of the skin diseases (*P* > .05) except for the diagnosis of malignant melanoma and basal cell carcinoma. While all family medicine residents registered in the CFMSTP (100%, n = 67) experienced difficulty or inadequacy in making the diagnosis of malignant melanoma, this rate was 90.5% (n = 38) among general practitioners, 94.9% (n = 37) among family medicine specialists, and 82.1% (n = 23) in family medicine residents of MSP. The differences were statistically significant (Fisher Exact Test, *P* = .003). Additionally, while all of the family medicine residents registered in the CFMSTP (100%, n = 67) experienced difficulty or inadequacy in the diagnosis of basal cell carcinoma, this rate was 90.5% (n = 38) among general practitioners, 89.7% (n = 35) among family medicine specialists, and 92.9% (n = 26) among family medicine residents of MSP. A statistically significant difference was found between the job positions of the family physicians (Fisher Exact Test, *P* = .020).

The participants’ competencies in treating different dermatological diseases were also analyzed according to their medical specialization status. Statistically significant differences were found between medical specialization statuses only in terms of cellulite/erysipelas and acne treatment. Three percent (n = 2) of family medicine residents registered in the CFMSTP, 14.3% (n = 6) of general practitioners, 15.4% (n = 6) of family medicine specialists, and 35.7% (n = 10) of family medicine residents employed in the MSP declared self-inadequacy or lack of confidence in acne treatment (Pearson chi-square = 18.160, *P* < .001). On the other hand, 17.9% (n = 12) of family medicine residents registered in the CFMSTP, 26.2% (n = 11) of general practitioners, 7.7% (n = 3) of family medicine specialists, and 3.6% (n = 1) of family medicine residents registered in MSP stated self-inadequacy or uncertainty in cellulite/erysipelas treatment (Pearson chi-square = 8.890, *P* = .031).

## Discussion

Our study’s results show that family physicians deal with a high rate (median: 13%) of skin diseases in the outpatient clinics within a month. Despite this high rate, the majority of the participants reported to face difficulty or incompetence while diagnosing and treating fundamental skin diseases such as malignant melanoma, squamous cell carcinoma, basal cell carcinoma, and hidradenitis suppurativa. Additionally, the status of completing a clinical dermatology rotation was found to have a statistically significant relationship with the diagnostic and therapeutic competence and self-confidence of the participants. These findings highlight the importance of clinical dermatology education and practice that family physicians complete during their training period. When compared to other studies from Türkiye, this study investigated both the diagnostic and therapeutic approaches of the family physicians to different skin diseases in relation to the completion of dermatology rotation status and medical specialization status. Therefore, it was believed that the present study sheds light upon the significance of dermatologic training during medical education.

Another study performed in Türkiye by Özyurt et al^3^ investigated the knowledge of family physicians regarding commonly seen skin disorders, as well as their diagnostic and therapeutic modalities. In this study, high rates of inaccurate management modalities for nail and bacterial skin diseases were observed among the family physicians.^3^ The majority of the participants stated that they did not have any difficulty in the management of fungal skin diseases, urticaria, allergic skin diseases, acne vulgaris, and bacterial/viral skin diseases, whereas 47.3% of the participants found psoriasis and lichen planus diseases challenging to manage.^3^ Similarly, in this study, fungal skin infections, seborrheic dermatitis, acne, atopic dermatitis, herpes simplex infection, and viral warts were among the cutaneous diseases in which the majority of the family physicians felt confident and successful while treating. It was believed that high rates of admission to the outpatient clinics due to fungal skin infections, acne vulgaris, herpes simplex infections, and different types of dermatitis, as shown in this study, might have enabled the physicians to develop their management skills and clinical experience in these diseases.

Another survey-based study from Türkiye that investigated the dermatological practice of primary care doctors and their thoughts about undergraduate medical education showed that eczema, fungal skin infections, urticaria, acne, and psoriasis were the most frequent skin diseases encountered by primary care physicians.^2^ Supporting these findings, mycoses affecting the skin, hair, nail and mucous membranes; acne; atopic dermatitis and seborrheic dermatitis were among the most common skin disorders seen by family physicians during their medical practice. It was believed that the high prevalance rates of atopic dermatitis,^4^ fungal and viral skin infections,^5^ and acne vulgaris^6^ in Türkiye might account for the elevated number of patients who applied for these skin conditions. In the same study by Aybal et al^2^ primary care physicians reported to face difficulty while making the diagnosis of skin neoplasms, Behçet’s disease, parasitic skin infections, and rosacea. In this study, it was found that the majority of the family physicians experienced difficulty or felt incompetent while diagnosing cutaneous neoplasms such as squamous cell carcinoma, basal cell carcinoma, and malignant melanoma along with hidradenitis suppurativa and psoriasis. Since the diagnoses of various skin neoplasms require a higher degree of clinical suspicion, comprehensive theoretical dermatologic education, including dermoscopic training and confirmation by histopathology, primary care physicians are most likely to refer patients to a dermatology specialist. For example, a questionnaire-based study from the USA which analyzed the dermoscopy practice among family physicians showed that only 16% of participants reported having access to a dermatoscope, only 15% had dermoscopy training, and only 9% used a dermatoscope regularly in their daily practice.^7^ Low rates of dermoscopy knowledge and usage might have led family physicians in Türkiye to face difficulty while diagnosing skin tumors as well.

In their study, Aybal et al^2^ also found that superficial fungal infections, burns, and acne vulgaris were the 3 most common skin conditions that primary care physicians treated themselves, whereas skin neoplasms, refractory pruritus, and erythematous scaly disease were the main disease categories that they felt the necessity to refer to a specialist. Similarly, malignant skin neoplasms (basal cell carcinoma, squamous cell carcinoma, and malignant melanoma) and psoriasis were some of the cutaneous diseases at which family physicians felt incompetent while treating. It was believed that malignant skin tumors and psoriasis might have been well-accepted by family physicians as specific diseases of dermatology that require precise approaches and management by dermatologists.

In Türkiye, both theoretical and practical dermatology training are generally completed in the fifth grade of medical faculty. Additionally, for those who undergo family medicine specialization training, dermatology rotation provides an opportunity for family medicine residents to develop their clinical, diagnostic, and therapeutic skills related to various skin disorders. As the main coordinators of primary care, family physicians play an important role in triaging patients and reducing the necessity for specialized care, including dermatology.^8^ Therefore, there is an ever-increasing expectation on family physicians to support and sustain care for patients with frequently seen skin problems.^8^^-^^10^ In order to meet this expectation, both vocational undergraduate training courses and continuous medical education programs should be fortified.^11^ This study showed that 47.2% of all family doctors included had completed a clinical dermatology rotation during their training, and 62.7% of those who completed the rotation found it beneficial and efficient. Consequently, statistically significant differences were found between the groups who had a dermatology rotation and those who did not, in terms of diagnostic competence (for scabies and drug eruption) and therapeutic proficiency (for atopic eczema, cellulitis/erysipelas, and scabies). In line with this result, several studies reported improvement in clinical practice skills and enhanced patient care after completing postgraduate training courses.^12^^,^^13^Another study from Florence, Italy, which examined the diagnostic precision and referral threshold of family physicians for melanoma after formal training, found that the rate of accurately diagnosed melanoma cases significantly increased after the training.^14^ This study highlights the positive impact of continuing medical education on the clinical approach to suspicious skin lesions. Furthermore, Raash et al^15^ reported another study in which family physicians were randomly selected for an educational intervention related to the management of doubtful skin neoplasms or a control group. In the intervention group, improved clinical data on pathology requests and the family physician’s diagnostic confidence were observed.^15^ Another study letter from Türkiye by Ozcan et al^11^ which evaluated the efficiency of postgraduate dermatologic education, has drawn attention to the need for re-evaluation and consolidation of undergraduate dermatologic training. A cross-sectional assessment study from Western India highlighted the poor knowledge of primary care physicians in diagnosing and managing dermatologic diseases after performing a pre-validated, self-rating questionnaire and photo-quiz related to skin disorders.^16^ In a systematic review by Nawal et al^17^, it was shown that family physicians deal with a wide range of skin disorders in their daily practice, and reinforcement of dermatologic training may improve diagnostic and therapeutic management skills.

All in all, even though this study has limitations due to the limited number of participants and survey-based design, the results show that family physicians deal with a considerable number of patients with skin problems in the outpatient clinics in Türkiye.

In recent years, there seems to be a global trend that aims to ameliorate the quality of primary care by improving clinical skills, diagnostic and therapeutic proficiency through postgraduate medical education. In line with the results of this study, the importance of qualified, effective, and intense practical and theoretical undergraduate training, dermatology rotation during family medicine specialization, and continuing medical education in enhancing primary dermatologic care in Türkiye are also highlighted.

## Figures and Tables

**Table 1.** Demographical, Occupational and Educational Characteristics of the Family Physicians

Sociodemographic and Occupational Characteristics of Family Physicians	Median (Minimum-Maxium)
Age (years)	35 (26-67)
Time duration for working as a family physician (years)	7 (0.25-29)
Average number of patients examined in a month	1025 (90-4000)
% of patients examined within a month due to skin problems	13 (1-90)
% of patients applied for skin problems and diagnosed&treated by the family physician in a month	75 (1-100)
**Gender**	**Frequency (%)**
Male	78 (44.3)
Female	98 (55.7)
**Healthcare facilities in which the family physicians worked**	**Frequency (%)**
Family health center	120 (68.2)
Research and training hospital	20 (11.4)
State hospital	18 (10.2)
University hospital	7 (4)
District health directorate/community health center	7 (4)
Others	4 (2.2)
**Position as a family physician**	**Frequency (%)**
Family medicine resident registered to ‘Contractual Family Medicine Specialization Training Program’	67 (22.2)
Practitioner	42 (23.9)
Family medicine specialist	39 (38.1)
Family medicine resident in ‘Medical Speciality Program’	28 (15.9)
**Status of completing a clinical dermatology rotation**	**Frequency (%)**
Did not complete any dermatology rotation	51 (29)
Completed a dermatology rotation	83 (47.1)
Those without any specialization training	42 (23.9)

**Table 2.** Diagnostic Confidence and Difficulty Levels of the Family Physicians with Respect to Different Skin Diseases

Skin Disease Type	Have No Knowledge Related To Diseasen (%)	Know the Clinical Features of the Disease, Cannot Make a Diagnosisn (%)	Make the Diagnosis with Difficulty, But Not with Full Confidencen (%)	Make the Diagnosis with Some Difficulty, But with Confidencen (%)	Make the Diagnosis Confidentlyn (%)
Acne	0 (0)	3 (1.7)	6 (3.4)	43 (24.4)	124 (70.5)
Allergic-irritant contact dermatitis	0 (0)	1 (0.6)	17 (9.7)	74 (42)	84 (47.7)
Alopecia areata	0 (0)	10 (5.7)	20 (11.4)	48 (27.3)	98 (55.7)
Atopic dermatitis	1 (0.6)	10 (5.7)	28 (15.9)	71 (40.3)	66 (37.5)
Basal cell carcinoma	15 (8.5)	116 (65.9)	35 (19.9)	7 (4)	3 (1.7)
Fungal infections of skin, hair, nail and mucous membranes	0 (0)	0 (0)	14 (8)	50 (28.4)	112 (63.6)
Herpes simplex infection	0 (0)	1 (0.6)	5 (2.8)	29 (16.5)	141 (80.1)
Hidradenitis suppurativa	22 (12.5)	24 (13.6)	54 (30.7)	51 (29)	25 (14.2)
Drug eruption	5 (2.8)	20 (11.4)	71 (40.3)	52 (29.5)	28 (15.9)
Malignant melanoma	9 (5.1)	121 (68.8)	35 (19.9)	8 (4.5)	3 (1.7)
Seborrheic dermatitis	0 (0)	4 (2.3)	17 (9.7)	58 (33)	97 (55.1)
Psoriasis	0 (0)	38 (21.6)	57 (32.4)	44 (25)	37 (21)
Cellulitis/erysipelas	0 (0)	3 (1.7)	27 (15.3)	64 (36.4)	82 (46.6)
Scabies	0 (0)	1 (0.6)	36 (20.5)	77 (43.8)	62 (35.2)
Squamous cell carcinoma	17 (9.7)	119 (67.6)	30 (17)	8 (4.5)	2 (1.1)
Viral warts	0 (0)	6 (3.4)	30 (17)	58 (33)	82 (46.6)
Zona Zoster	0 (0)	1 (0.6)	6 (3.4)	45 (25.6)	124 (70.5)

**Table 3.** Therapeutic Approaches of the Family Physicians to Variable Skin Disorders

**Skin Disease Type**	Do Not Start Any Treatment, Refer the Patient to the Relevant Specialist**n (%)**	Administer the Treatment with No Confidence**n (%)**	Administer the Treatment with Little Confidence**n (%)**	Administer the Treatment with Full Confidence**n (%)**
Acne	18 (10.2)	6 (3.4)	54 (30.7)	98 (55.7)
Allergic-irritant contact dermatitis	4 (2.3)	7 (4)	69 (39.2)	96 (54.5)
Alopecia areata	75 (42.6)	19 (10.8)	42 (23.9)	40 (22.7)
Atopic dermatitis	17 (9.7)	16 (9.1)	56 (31.8)	87 (49.4)
Basal cell carcinoma	171 (97.2)	3 (1.7)	2 (1.1)	0 (0)
Fungal infections of skin, hair, nail and mucous membranes	2 (1.1)	1 (0.6)	35 (19.9)	138 (78.4)
Herpes simplex infection	0 (0)	1 (0.6)	36 (20.5)	139 (79)
Hidradenitis suppurativa	79 (44.9)	20 (11.4)	49 (27.8)	28 (15.9)
Drug eruption	49 (27.8)	22 (12.5)	58 (33)	47 (26.7)
Malignant melanoma	170 (96.6)	3 (1.7)	1 (0.6)	2 (1.1)
Seborrheic dermatitis	11 (6.3)	7 (4)	61 (34.7)	97 (55.1)
Psoriasis	87 (49.4)	17 (9.7)	44 (25)	28 (15.9)
Cellulitis/erysipelas	20 (11.4)	7 (4)	65 (36.9)	84 (47.7)
Scabies	3 (1.7)	7 (4)	43 (24.4)	123 (69.9)
Squamous cell carcinoma	172 (97.7)	2 (1.1)	2 (1.1)	0 (0)
Viral warts	61 (34.7)	8 (4.5)	49 (27.8)	58 (33)
Zona Zoster	10 (5.7)	3 (1.7)	37 (21)	126 (71.6)

## Data Availability

The data that support the findings of this study are available on request from the corresponding author.
